# Prediction of Alzheimer's disease using individual structural connectivity networks

**DOI:** 10.1016/j.neurobiolaging.2012.01.017

**Published:** 2012-12

**Authors:** Junming Shao, Nicholas Myers, Qinli Yang, Jing Feng, Claudia Plant, Christian Böhm, Hans Förstl, Alexander Kurz, Claus Zimmer, Chun Meng, Valentin Riedl, Afra Wohlschläger, Christian Sorg

**Affiliations:** a Department of Neuroradiology of Klinikum rechts der Isar, Technische Universität München, Ismaningerstrasse 22, 81675 Munich, Germany; b Institute for Computer Science, Ludwig-Maximilians-Universität München, Oettingenstraβe 67, 80538 Munich, Germany; c Department of Psychiatry of Klinikum rechts der Isar, Technische Universität München, Ismaningerstrasse 22, 81675 Munich, Germany; d Department of Experimental Psychology, Oxford University, South Parks Road, OX1 3UD Oxford, UK; e School of Engineering, University of Edinburgh, Mayfield Road, EH9 3JL Edinburgh, UK; f General and Experimental Psychology/Neuro-Cognitive Psychology, Ludwig-Maximilians-Universität München, Leopoldstrasse 13, 80802 Munich, Germany; g Department of Nuclear Medicine of Klinikum rechts der Isar, Technische Universität München, Ismaningerstrasse 22, 81675 Munich, Germany

**Keywords:** Alzheimer's disease, Structural connectivity, Diffusion tractography, Classification

## Abstract

Alzheimer's disease (AD) progressively degrades the brain's gray and white matter. Changes in white matter reflect changes in the brain's structural connectivity pattern. Here, we established individual structural connectivity networks (ISCNs) to distinguish predementia and dementia AD from healthy aging in individual scans. Diffusion tractography was used to construct ISCNs with a fully automated procedure for 21 healthy control subjects (HC), 23 patients with mild cognitive impairment and conversion to AD dementia within 3 years (AD-MCI), and 17 patients with mild AD dementia. Three typical pattern classifiers were used for AD prediction. Patients with AD and AD-MCI were separated from HC with accuracies greater than 95% and 90%, respectively, irrespective of prediction approach and specific fiber properties. Most informative connections involved medial prefrontal, posterior parietal, and insular cortex. Patients with mild AD were separated from those with AD-MCI with an accuracy of approximately 85%. Our finding provides evidence that ISCNs are sensitive to the impact of earliest stages of AD. ISCNs may be useful as a white matter-based imaging biomarker to distinguish healthy aging from AD.

## 1 Introduction

Alzheimer's disease (AD), the most common cause of age-related dementia, is a neurodegenerative disease characterized by increasing cognitive and behavioral deficits (Blennow et al., 2006). AD is neuropathologically characterized by amyloid plaques, neurofibrillary tangles, and the loss of neurons, with changes starting regionally and spreading out gradually across the brain's gray matter (Braak and Braak, 1991; Thal et al., 2002). In addition, postmortem histological and *in vivo* imaging studies demonstrate widespread alterations of patients' white matter (Bozzali et al., 2002; Brun and Englund, 1986a; Rose et al., 2000). These reports of spreading cell loss and white matter deterioration have motivated the hypothesis that the cognitive and behavioral symptoms of AD are the consequence of disconnection between brain regions (Brun and Englund, 1986b; Delbeuck et al., 2003; Lo et al., 2010; Sorg et al., 2009). Therefore, we hypothesized that subject-specific patterns of changed white matter connectivity reflect the emergence of observable deficits in behavior and cognition.

To this end, we used diffusion-weighted magnetic resonance imaging (DWI) to explore white matter microstructure *in vivo* (Johansen-Berg and Rushworth, 2009). During DWI, multiple brain images sensitive to different water diffusion directions were acquired, and, subsequently, data were fitted to a mathematical diffusion tensor model for each voxel. The diffusion tensor model describes diffusion as an ellipsoid and allows for both the identification of local diffusion properties (such as fractional anisotropy [FA] or mean diffusivity [MD]) and the diffusion-based reconstruction of fiber paths (Lazar et al., 2003; Mori et al., 1999). Local FA and MD values reflect the local density and integrity of fiber bundles voxel by voxel. By contrast, fiber paths reflect the density and integrity of long-range connections between cortical regions, possibly making them a better correlate of cortical information processing and, hence, cognitive function (Johansen-Berg and Rushworth, 2009).

DWI studies in AD have demonstrated aberrant FA and MD values in the white matter of the frontal, occipital, and temporal lobes (Bozzali et al., 2002; Fellgiebel et al., 2004; Sexton et al., 2010; Stahl et al., 2007), as well as in selected tracts such as the corpus callosum or cingulum bundle (Fellgiebel et al., 2005; Liu et al., 2009a; Sexton et al., 2010; Stahl et al., 2007). DWI-based studies in mild cognitive impairment (MCI), which is a high-risk state for AD, have demonstrated similarly distributed, but less severe, FA/MD changes (Fellgiebel et al., 2004, 2005; Stahl et al., 2007). Recently, DWI-based tractography (Lo et al., 2010) has indicated that AD dementia leads to changes in the topological organization of individual, fiber-based, structural connectivity networks, and that these changes correlate with cognitive deficits.

Here, we asked (i) whether individual structural connectivity networks (ISCNs) based on DWI tractography are already changed in predementia forms of AD and (ii) whether ISCNs can be used to distinguish individual patients with predementia or mild AD from healthy control subjects (HC). Patients with predementia AD were defined by MCI at the time of the DWI scan and conversion to AD dementia within 3 years (AD-MCI) (Table 1). For each subject, diffusion tractography and 96 predefined cortical regions were used to construct ISCNs. We then extracted three attributes (fiber density, FA, and MD) for each connection. ISCN patterns were finally used to predict the clinical status of subjects by applying machine learning-based pattern recognition techniques (Klöppel et al., 2008; Plant et al., 2010).

## 2 Methods

### 2.1 Subjects

Seventeen patients with mild AD (range, 55 to 83 years with an average of 68.9 ± 8.1 years; 7 female), 23 patients with AD-MCI (range, 59 to 79 years with an average of 67.6 ± 5.4 years; 11 female), and 21 healthy control subjects (range, 56 to 85 years with an average of 66.3 ± 7.4 years; 13 female) participated in this study (see Table 1). All participants provided informed consent in accordance with the Human Research Committee guidelines of the Klinikum Rechts der Isar, Technische Universität (Munich, Germany). Patients were recruited from the Memory Clinic of the Department of Psychiatry, and healthy control subjects were recruited by word-of-mouth advertising. Examination of every participant included medical history, neurological examination, neuropsychological assessment (Consortium to Establish a Registry for Alzheimer's Disease [CERAD]) (Morris et al., 1989), structural MRI, and (for patients only) informant interview (Clinical Dementia Rating [CDR]) (Morris, 1993), as well as blood tests. Patients with mild AD fulfilled criteria for dementia (CDR global score = 1) and the National Institute of Neurological Disorders and Stroke–Alzheimer's Disease and Related Disorders Association (NINCDS-ADRDA) criteria for AD (McKhann et al., 1984). Patients with AD-MCI met criteria for amnestic MCI at baseline and converted to mild AD within 3 years. Amnestic MCI criteria include reported and neuropsychologically assessed memory impairments, largely intact activities of daily living, and excluded dementia (CDR = 0.5) (Gauthier et al., 2006). Conversion to AD was assessed during annual follow-up clinical assessments after baseline, including medical history, neurological examination, informant interview (CDR), and neuropsychological assessment (CERAD). Four patients converted to AD after 1 year, 7 after 2 years, and 12 after 3 years. Exclusion criteria for entry into the study were other neurological, psychiatric, or systemic diseases (e.g. stroke, depression, alcoholism) or clinically remarkable MRI (e.g. stroke lesions) potentially related to cognitive impairment. A total of 8/12/10 persons with mild AD/AD-MCI/control subjects were treated for hypertension (beta-blockers, ACE inhibitors, and calcium-channel blockers), and 7/7/9 were treated for hypercholesterolemia (statins). In all, 3/1/0 individuals had diabetes mellitus, 2/3/0 received antidepressant medication (mirtazapine, escitalopram), and all patients with mild AD received cholinesterase inhibitors. None of the control subjects were taking any psychotropic medication.

### 2.2 Data acquisition

On a 3-T MRI scanner (Achieva, Philips, The Netherlands), we acquired DWI using a pulsed gradient spin-echo echo planar imaging sequence with a parallel imaging (SENSE) factor of 2.5, echo time (TE) = 60 ms, and repetition time (TR) = 6516 ms. Images were acquired for a 112 × 112-matrix size of slice and subsequently reconstructed for a 128 × 128-matrix size, with a resolution of 1.75 mm in plane and a slice thickness of 2 mm. A total of 60 contiguous slices were acquired to give complete brain coverage containing 128 × 128 × 60 voxels with a size of 1.75 × 1.75 × 2 mm^3^. Diffusion gradients were applied in 15 noncollinear directions with b = 800 s/mm^2^. B0 image without diffusion weighting, b = 0 s/mm^2^, was additionally acquired.

### 2.3 Construction of ISCNs

**Fig. 1:**
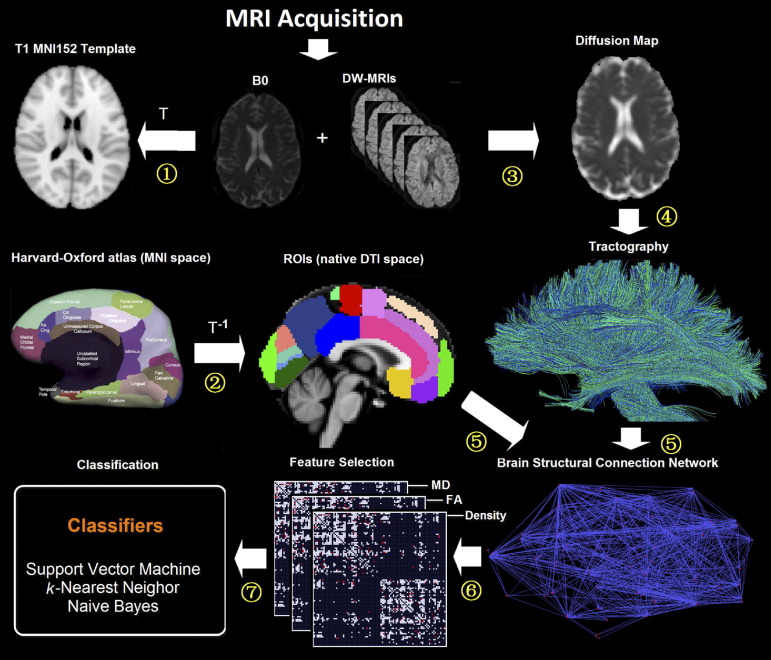
Flowchart of DWI image analysis. (1) Individual non-DWI (B0) images were affine-registered to the ICBM 152 template of Montreal Neurological Institute space to obtain the transformation matrix (T) for each participant. (2) The inverse transformation matrix (T^−1^) was then applied to both Harvard-Oxford brain atlas and (B0) image to generate corresponding cerebral regions in each individual's DWI native space. (3) After preprocessing of DWIs, the local properties of water diffusion (e.g. fractional anisotropy [FA] or mean diffusivity [MD]) were derived from the voxel-wise diffusion tensor model. (4) Whole brain tractography was performed providing an estimate of axonal trajectories across the entire white matter. (5) Individual structural connectivity networks (ISCNs) were constructed by combining the output of both cortical parcellation and diffusion tractography for each individual subject. (6) The most distinctive connections of ISCNs among groups were identified by a feature selection criterion for different attributes of fiber density, FA, and MD. (7) ISCNs of patients with mild cognitive impairment and mild Alzheimer's disease (AD), respectively, and healthy control subjects were classified by three different pattern recognition algorithms.

To construct the structural connectivity network for each participant, the procedure involved the following steps (Fig. 1).

Cortical parcellation. For cortical parcellation, the whole brain of each participant was segmented into 96 cortical regions using the Harvard-Oxford cortical structural atlas (http://www.fmrib.ox.ac.uk/fsl). This anatomical atlas is a probabilistic population-based atlas; subregions were thresholded in a way that only voxels, which are estimated above 35% probability of being in that structure, are included in the mask. We used 35% as a threshold value to prevent overlap between regions of interest (ROIs) after registering them to the B0 images of individual participants. This also increased the likelihood of capturing fibers originating from the center of a region, where variability across individuals is smallest. Each individual's B0 image was first affine-registered (with 12 degrees of freedom) to the ICBM 152 template of the Montreal Neurological Institute space (MNI, http://www.bic.mni.mcgill.ca) to obtain the transformation matrix (T). The inverse transformation matrix (T^−1^) was then applied to the Harvard-Oxford atlas to generate corresponding cortical regions in each individual's DWI (B0) native space.

Diffusion tractography. To determine the connectivity between pair-wise regions, diffusion tractography was used. Distortion induced by motion for weighted MRI images was first corrected by aligning all DWIs to the non-DWI (B0) using a 2-D linear registration algorithm (automated image registration) (Woods et al., 1998). Maps of the diffusion tensor elements, MD, and FA and major eigenvector direction were calculated for each voxel by using in-house software. Then the deterministic fiber tracking algorithm TEND (Lazar et al., 2003) was applied to investigate the brain's white matter for each subject. Because voxels with high FA are more likely to contain a high proportion of white matter, all voxels with FA > 0.3 were selected as seed points of fiber tracking (Lazar et al., 2003; Liu et al., 2009b; Mori et al., 1999). Selecting seed voxels with FA > 0.3 ensures the trajectories originated from the white matter tissue, and potential possible trajectories can be successfully reconstructed. Tracking started from these seed points, using the major eigenvectors of seed points as the original propagation directions. Then tracts are propagated by using the entire diffusion tensor to deflect the estimated fiber trajectory in both directions. Tracking stopped in voxels with FA < 0.2 or physiologically implausible curvature of the track (> 60°) (Lazar et al., 2003; Liu et al., 2009b; Mori et al., 1999).

**Fig. 2:**
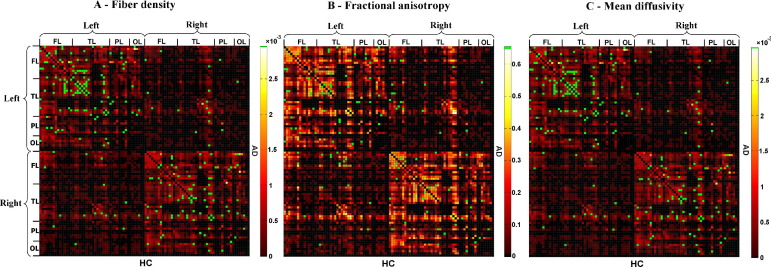
Selected connections in ISCNs for the comparison between patients with mild AD and healthy control subjects using information gain criterion. The three matrices represent the averaged structural connectivity network based on different attributes: (A) fiber density, (B) fractional anisotropy (FA), and (C) mean diffusivity. Each element of the matrix represents the connection between two cortical regions. In each matrix, the upper triangle matrix indicates the averaged structural connectivity for patients with mild AD, and the lower triangle matrix indicates the averaged structural connectivity for healthy control subjects. Black dots indicate that there is no connection for any subject of the group. Red to yellow dots indicate the average of connection attribute across all subjects of the group (yellow indicates higher scores). Note that connection attribute is defined by the mean of, for example, FA values across all voxels of fibers constituting the connection in one subject. Green dots in each matrix indicate the discriminative connections for group comparison selected by information gain criterion in more than 5 rounds of 10-fold cross-validation. FL refers to frontal lobe; TL, temporal lobe; PL, parietal lobe; OL, occipital lobe. For interpretation of the references to color in this figure legend, the reader is referred to the Web version of this article.

**Fig. 3:**
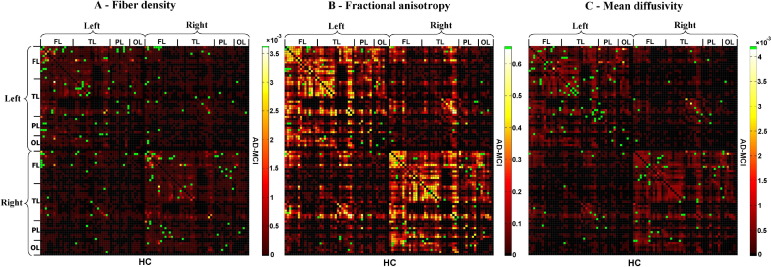
Selected connections in ISCNs for the comparison between patients with AD-MCI and healthy control subjects using information gain criterion. The three matrices represent the averaged structural connectivity network based on different attributes: (A) fiber density, (B) fractional anisotropy (FA), and (C) mean diffusivity. Each element of the matrix represents the connection between two cortical regions. In each matrix, the upper triangle matrix indicates the averaged structural connectivity for patients with AD-MCI, and the lower triangle matrix indicates the averaged structural connectivity for healthy control subjects. Black dots indicate that there is no connection for any subject of the group. Red to yellow dots indicate the average of connection attribute across all subjects of the group (yellow indicates higher scores). Note that connection attribute is defined by the mean of, for example, FA values across all voxels of fibers constituting the connection in one subject. Green dots in each matrix indicate the discriminative connections for group comparison selected by information gain criterion in more than 5 rounds of 10-fold cross-validation. FL refers to frontal lobe; TL, temporal lobe; PL, parietal lobe; OL occipital lobe. For interpretation of the references to color in this figure legend, the reader is referred to the Web version of this article.

**Fig. 4:**
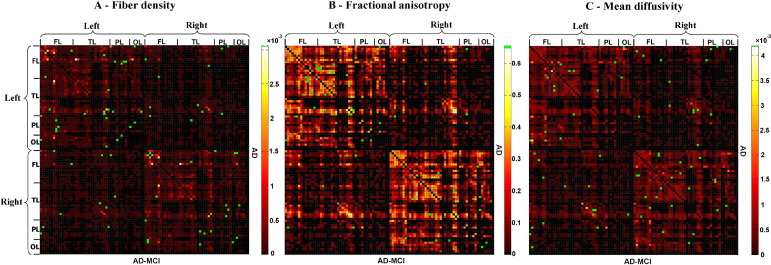
Selected connections in ISCNs for the comparison between patients with mild AD and AD-MCI using information gain criterion. The three matrices represent the averaged structural connectivity network based on different attributes: (A) fiber density, (B) fractional anisotropy, and (C) mean diffusivity. Each element of the matrix represents the connection between two cortical regions. In each matrix, the upper triangle matrix indicates the averaged structural connectivity for patients with mild AD, and the lower triangle matrix indicates the averaged structural connectivity for patients with AD-MCI. Black dots indicate that there is no connection for any subject of the group. Red to yellow dots indicate the average of connection attribute across all subjects of the group (yellow indicates higher scores). Note that connection attribute is defined by the mean of, for example, FA values across all voxels of fibers constituting the connection in one subject. Green dots in each matrix indicate the discriminative connections for group comparison selected by information gain criterion in more than 5 rounds of 10-fold cross-validation. FL refers to frontal lobe; TL, temporal lobe; PL, parietal lobe; OL, occipital lobe. For interpretation of the references to color in this figure legend, the reader is referred to the Web version of this article.

ISCN construction. The output of both cortical parcellation and diffusion tractography was combined to construct individual structural connectivity networks ISCNs for each subject. Each atlas-based region was regarded as a network node. Connectivity of each pair of nodes was measured by fibers across two regions. If there exists at least one fiber with end points in one pair of regions (e.g. region *i* and region *j*), the two cortical regions are assumed to be connected (Hagmann et al., 2007, 2008). To control for the influence of this definition of connection, we performed the same analyses also for connection definitions based on three and five fibers (e.g. Lo et al., 2010) (Tables S4 and S5). For each connection, three attributes were calculated: (a) fiber density *cd*_*ij*_ of a connection was defined as proportion of all fibers connecting the two regions (*n*_*ij*_) over the total number of fibers of the subject (*n*_*all*_), that is, *cd*_*ij*_ *= n*_*ij*_/*n*_*all*_ (Hagmann et al., 2007, 2008; Roberts et al., 2005). (b) *FA*_*ij*_ of a connection was defined as the mean value of FA across all voxels of all connection fibers. (c) *MD*_*ij*_ of a connection was defined as the mean value of MD across all voxels of connection fibers. Each attribute reflects the weighted edge of a network, that is, finally three different ISCNs defined by patterns of *cd*_*ij*_, *FA*_*ij*_, and *MD*_*ij*_ were obtained for each subject. Figures 2–4 display connectivity matrices that reflect connection attributes averaged across subjects for each group and attribute.

### 2.4 Pattern classification of ISCNs

After construction of ISCNs, individual connectivity patterns were classified by the use of three different machine learning-based pattern classifiers together with a feature selection procedure (Figs. 2–4). To reduce variability of results and to evaluate how results generalize to an independent data set, 10-fold cross-validation (Table 2) and leave-one-out validation (Table S3) were applied (Mosteller, 1948). For each type of ISCN, for all possible between-group comparisons, and for each round of validation, first feature selection (on the training data) using an information gain criterion (Quinlan, 1993) was used to rate the information-based interestingness of each connection and attribute to distinguish groups (Supplementary Data [SD], Table S2). After that, three classifiers, namely, support vector machine (SVM), *k*-nearest neighbor (*k*-NN), and naive Bayes (NB) (see later in the text), were used to classify and predict subjects based on selected connections. Classification results were averaged over rounds of validation. In Figures 2–4, green dots indicate connections that were selected by information gain in more than five rounds of validation, suggesting their relevance for group separation (Figs. 2–4).

The basic idea of SVM procedures (Vapnik, 1995) is to construct a separating hyperplane between the training instances of both classes (groups). Among all possible hyperplanes, the one with the maximum margin between classes is selected. Given *m* training vectors *x*_*k*_*∈R*^*n*^ (*k* = 1,…, *m*) of two classes, and a vector of labels *y∈R*^*m*^ such that *y*_*k*_*∈*{1, −1}, then SVM solves a quadratic optimization problem:

(1) $$\underset{w,b,ɛ}{\min}\frac{1}{2}\omega^{T}\omega +C\overset{m}{\underset{k=1}{\sum }}{ɛ}_{k}\;\;\;with\;y_{k}\left(\omega^{T}\varphi \left(x_{k}\right)+b\right)\geq 1-{ɛ}_{k},\;{ɛ}_{k}\geq 0,\;k=1,....,m,$$

where *ω* is a normal vector, *b* is a scalar, and *ε*_*k*_ are non-negative variables, *C* is a penalty parameter on the training error, *y*_*k*_ is the class label, and *φ*(·) is a map function to transfer the training data into a higher dimensional space. For any testing instance *x*, the critical decision function (predictor) then has the form:

(2) $$f\left(x\right)=\mathrm{sgn}\left(\omega^{T}\varphi \left(x\right)+b\right)$$

where *f* (*x*) is the prediction function, *sgn*(*·*) is the sign function, and ω^T^ is the transpose of normal vector *ω*.

*k*-NN (Aha et al., 1991) is a method for classifying objects based on closest training examples in the feature space. It is a typical instance-based learning algorithm, where an object is classified by a majority vote of its neighbors, with the object being assigned to the class most common among its *k*-NNs. Given an instance *x*, its *k*-NNs are found in terms of the Pearson correlation coefficient, and then its label value is determined by these *k* neighbors using the majority vote manner principle. In this study, we specify *k* = 6 for all experiments.

The NB classifier (Domingos and Pazzani, 1997) is a simple probabilistic classifier based on applying Bayes' theorem with strong (i.e. naive) independence assumptions. A NB classifier assumes that the presence/absence of a particular feature of a class is unrelated to the presence/absence of any other feature. The fundamental idea of Bayesian classification is to classify instances *x* based on a probability model, which is defined as:

(3) $$NB\left(x\right)=\underset{c\in C}{\arg\;\max}\;p\left(c|x_{1},\ldots ,x_{n}\right)$$

with classes C and feature variables *x*_*1*_*,…, x*_*n*_ constituting the components of *x*. Using Bayes' theorem, it can be rewritten as:

(4) $$NB\left(x\right)=\underset{c\in C}{\arg\;\max}\frac{p\left(c\right)p\left(x_{1},\ldots ,x_{n}|c\right)}{p\left(x_{1},\ldots ,x_{n}\right)}=\underset{c\in C}{\arg\;\max}\;p\left(c\right)p\left(x_{1},\ldots ,x_{n}|c\right)$$

Because NB assumes that the conditional probabilities of the independent variables are statistically independent, the prediction function of NB is finally defined as:

(5) $$NB\left(x\right)=\underset{c\in C}{\arg\;\max}\;p\left(c\right)\underset{i}{\prod }p(x_{i}|c).$$

## 3 Results

For each type of ISCN, different pattern classifiers and validation methods were used to estimate individual clinical group predictions. The following results are based on the use of SVM, 10-fold cross-validation, and connections based on at least one fiber between regions.

(i) AD-MCI vs. HC: AD-MCI subjects were distinguished from healthy control subjects with an accuracy of 97.73% when using fiber density (Table 2). For FA and MD, classification accuracies were 84.09% and 93.18%, respectively.
(ii) Mild AD vs. HC: SVM obtained fully correct predictions of 100% based on the attributes fiber density and MD; regarding FA, classification accuracy was 92.11%.
(iii) AD-MCI vs. mild AD: Between the patient groups of AD-MCI and mild AD, classification accuracies were 85%, 82.5%, and 85% for fiber density, FA, and MD, respectively.

Classification accuracy was comparably high for the other pattern classifiers and validation methods (Tables 2 and S3). For connection definitions based on three and five fibers, accuracies slightly decreased (on average about 5%) but were consistent among each other (Tables S4 and S5). A number of regions were commonly selected across analyses, including the left and right insula, bilateral middle temporal gyri, the superior parietal lobules and cuneus, and the frontopolar cortex (Table S6 and Fig. S5).

## 4 Discussion

In the current study, diffusion tractography-based ISCNs and pattern recognition were used to study white matter changes in very early stages of AD. Compared with healthy control subjects, ISCN patterns of patients with AD-MCI and mild AD were significantly altered with respect to fiber density and fiber integrity. Based on individual scans, ISCNs enabled the prediction of AD-MCI and mild AD with accuracies of about 90% and 95%, respectively.

### 4.1 ISCN as a new tool to describe AD

Previous DWI-based studies in AD and MCI have demonstrated widely distributed white matter changes (for review, Sexton et al. [2010]). A meta-analysis of 41 DWI studies (Sexton et al., 2010) revealed AD-related fiber deterioration (decreased FA or increased MD) in most cortical lobes. These changes, although smaller, were already apparent in MCI, suggesting that progressive damage to fiber connections begins in predementia stages of AD. Most DWI studies transformed FA or MD images into a standard space (Damoiseaux et al., 2009; Medina et al., 2006; Stahl et al., 2007), which tends to remove the large individual variability of fibers (Hagmann et al., 2008; Johansen-Berg and Rushworth, 2009; Van den Heuvel et al., 2009). This is desirable when measuring local statistics such as FA, but makes it harder to identify changes in specific fiber paths within a bundle. However, AD leads to atrophy and cell death in regionally specific populations and spreads in a characteristic pattern. Thus, it is likely that, at least early on, only fibers of specific connections are affected. Region-to-region fiber connections may then be more meaningful in characterizing a disconnection syndrome such as AD (Brun and Englund, 1986b; Delbeuck et al., 2003; Lo et al., 2010; Sorg et al., 2009), and are potentially more sensitive.

Using fiber tracts to describe AD is not entirely new. For instance, a previous study (Lo et al., 2010) derived graph theory-based topological scores from ISCNs to compare AD dementia with healthy control subjects with univariate statistical methods, that is, they used graph theory to convert ISCNs to a small set of values reflecting the systemic organization of the whole brain. In contrast, here we used the entire multivariate ISCN pattern to allow the use of pattern recognition techniques. These techniques evaluate the connectivity pattern as a whole instead of element by element. We used canonical machine learning-based pattern recognition procedures (SVM, *k*-NN, and NB), which have been applied successfully in MRI data analysis (Klöppel et al., 2008; Plant et al., 2010).

It is important to note that this multivariate pattern was derived from a relatively coarse anatomical parcellation (by dividing cortical gray matter into 96 anatomically separable regions according to the Harvard-Oxford atlas). This puts our parcellation on the same order as that of the AAL atlas (which also uses about 100 regions; see Zalesky et al. [2010]). It is difficult to extrapolate how classification would be affected by the use of more fine-grained parcellations (such as the ∼1000 regions used by Hagmann et al. [2008]). In our case, more fine-grained parcellations are unlikely to have improved classification, as the fiber networks provided by our imaging and fiber tracking approach were already sparse (see Fig. 2). Furthermore, we were interested in probing disruptions to long-range connectivity in early AD, whereas fine-grained parcellations with high-resolution imaging would most likely benefit the identification of more local structural connectivity.

### 4.2 Disruption of ISCNs in very early AD

Concerning group differences between healthy control subjects and AD-MCI and mild AD patients, all classification results for different ISCN-pattern types (fiber density, FA, MD) were largely independent from selected classifiers, evaluation methods, and connection definition, indicating that results reflect real group differences in ISCN patterns (Tables 2, S3–5). Furthermore, different ISCN features of fiber density, FA, and MD produce largely comparable accuracy scores for each possible group comparison, in line with previous voxel-/ROI-based results of comparable FA and MD changes in AD/MCI, and therefore suggesting that AD does not selectively impact on diffusion tensor properties (Tables 2 and S3).

(i) Mild AD: Patients with mild AD were separated from healthy control subjects with an accuracy of more than 95%. Studies using similar pattern recognition methods, but gray matter features (such as regional volume) instead of white matter ones, observed comparable separation results (Davatzikos et al., 2008; Klöppel et al., 2008; Plant et al., 2010; Teipel et al., 2007). For example, Klöppel et al. (2008) used T1-weighted gray matter images and SVM to separate pathologically verified AD patients from healthy control subjects with an accuracy of 96%. The congruence of this almost perfect separation of AD patients from healthy control subjects based once on gray matter and once on white matter features is in line with the disconnection hypothesis (Delbeuck et al., 2003). Our results complement these findings by demonstrating changes in local connectivity patterns.

(ii) AD-MCI: Patients with AD-MCI were separated from healthy control subjects with an accuracy of more than 90%. Our result is in line with previous findings of widely distributed FA decreases and MD increases in MCI (Sexton et al., 2010). Studies of MCI patients using similar pattern recognition methods for gray matter features such as tissue density found comparable separation results (Cuingnet et al., 2011; Davatzikos et al., 2008; Teipel et al., 2007). For example, Davatzikos et al. (2008) used gray matter density as well as a combination of both high-dimensional image analysis and pattern classification to separate MCI patients from healthy control subjects with 90% classification accuracy. In a recent study, Cuingnet et al. (2011) demonstrated that these high accuracy scores for the separation of predementia AD from healthy aging by T1-weighted gray matter properties are replicable but strongly depend on preprocessing procedures and applied classification methods. Interestingly, our data based on white matter ISCNs seem to be relatively independent of structural connectivity criteria and classification algorithms. Further studies are necessary to support this result in more detail and to relate explicitly classification of very early AD based on T1-weighted imaging and DWI.

The MCI patients assessed in our study all converted to AD dementia within 3 years. Because patients were not diagnosed with any other disease at the time of scan and in follow-up assessments, their deficits and brain changes at the time of scanning were very likely caused by AD. This indicates that AD already alters ISCNs substantially in the predementia stage. Furthermore, we found that groups of AD-MCI and mild AD patients were separated with an accuracy of about 85%, suggesting that ISCN changes increase during the course of AD. Unfortunately, our study gives no information how ISCNs of nonconverting MCI patients separate from healthy control subjects and patients with AD-MCI. Future studies have to explore whether selected tracts of ISCNs are preferentially affected along the course of AD, whether ISCN changes are accompanied by increasing changes of topological ISCN characteristics, and whether ISCNs separate converters from nonconverters with MCI.

The connections, which most frequently distinguished both groups from the healthy control subjects, included areas of the medial posterior occipito-parietal cortex (i.e. the superior parietal lobule and the cuneus), which have been shown to exhibit signs of early functional disconnection as well (Sorg et al., 2007, 2009). Connections to the frontal poles and middle temporal gyri were also selected often. These regions of the medial prefrontal and temporal cortex contribute to the default mode network, a set of regions that has been implicated in early AD in numerous modalities (Sorg et al., 2009; Sperling et al., 2010). Other selected features included the left and right insula, which may have captured fiber abnormalities originating in the anterior medial temporal lobes (Acosta-Cabronero et al., 2010). Because this study focused on using all pertinent information to identify patients, future studies will have to quantify how much these frequently selected regions contribute to classification.

### 4.3 ISCN as imaging biomarker

Findings of the current study suggest that ISCNs may have the potential of providing an imaging- and white matter-based biomarker for the distinction between healthy aging and very early AD. Our approach has three main features desirable for any biomarker. (i) ISCN patterns were directly and automatically derived from individual subject data. (ii) The pattern classifiers we used have an appropriate training-test structure, which fulfills criteria of a diagnostic tool and makes it possible to establish a large training set to improve diagnostic accuracy (Klöppel et al., 2008). (iii) The scanning protocol and data analysis of the current study were economical in many respects: scanning time of less than 5 minutes, only 15 gradient images per data set, use of the B0 image for cortical parcellation, and application of a deterministic streamlining approach for fiber tracking allowing for both fast calculation of ISCNs and maximal control of single steps of data analysis. As ISCN definition and classification of new test data are performed within several minutes without any user interaction, the time for the whole procedure is not a practically limiting factor for using ISCNs as a biomarker.

Like any other biomarker, we suspect that clinicians would use ISCN patterns in conjunction with existing biomarkers for early detection of AD (such as Pittsburgh compound B-positron emission tomography [PIB-PET], or amyloid-β-42 count in cerebrospinal fluid). In addition, ISCN patterns could be joined with other measures for multimodal classification (Ewers et al., 2011). Our focus on connectivity patterns could become especially useful in distinguishing incipient AD dementia from other dementias that affect different brain networks, such as frontotemporal lobar degeneration (Klöppel et al., 2008). Future studies need to address this potential. In that context, one should consider that clinical diagnosis standards for neurodegenerative dementias include a significant risk for misdiagnosing in comparison with definitive neuropathological criteria. That is, ISCNs might be optimally evaluated for their biomarker potential in pathologically verified AD patients (Klöppel et al., 2008). However, as we confirmed the diagnosis of AD in our sample by several ways (such as careful exclusion of other causes like vascular diseases or other neurodegenerative diseases, AD compatibility of atrophy pattern in T1-weighted images, and clinical follow-up assessments), we suggest a high rate of valid diagnoses for our patient sample. Therefore, we believe that obtained high accuracy scores of AD classification based on ISCN patterns reflect the impact of AD on brain's white matter. As a last point to consider, ISCNs' biomarker potential and the replicability of our high classification rate (>90%) need to be studied using larger cohorts, different imaging protocols, and different MRI scanners.

## Disclosure statement

All authors certify that they do not have any actual or potential conflicts of interest, including any financial, personal, or other relationships with other people or organizations within 3 years of beginning this work, which could inappropriately influence (bias) this work.

We confirm that this submission is not under review at any other publication, and all co-authors have seen and agree with the contents of the manuscript.

## Figures and Tables

**Table 1:** Demographical and neuropsychological scores of patients and healthy control subjects

Group	HC (n = 21)	Mild AD (n = 17)	AD-MCI (n = 23)	*p*
Female	13	7	11	0.42
Male	8	10	12	
Age	66.4 ± 7.5	68.9 ± 8.1	67.6 ± 5.4	0.53
MMSE score	29.4 ± 0.8	22.1 ± 4.3	26.8 ± 2.0	<0.01
Delayed recall (CERAD)	6.5 ± 2.1	0.9 ± 1.8	3.1 ± 2.0	<0.01

Key: AD, Alzheimer's disease; HC, healthy controls; MCI, mild cognitive impairment; AD-MCI, MCI at baseline with conversion to AD within 3 years; MMSE, Mini-Mental State Examination; CERAD, Consortium to Establish Registry for Alzheimer's Disease; *p*, *p* value. For statistical evaluation of group differences, χ^2^ (gender) and ANOVA (age, MMSE, delayed recall) were used.

**Table 2:** Classification accuracy for individual structural connectivity networks using 10-fold cross-validation

	SVM	*k*-NN	Naive Bayes
Mild AD vs. HC			
Fiber density	100.0%	94.74%	100.0%
FA	92.11%	94.74%	100.0%
MD	100.0%	94.74%	89.47%
Mild AD vs. AD-MCI			
Fiber density	85.00%	85.00%	95.00%
FA	82.50%	75.00%	85.00%
MD	85.00%	82.50%	90.00%
AD-MCI vs. HC			
Fiber density	97.73%	81.82%	95.45%
FA	84.09%	88.64%	97.73%
MD	93.18%	86.36%	100.0%

Key: SVM, support vector machine; *k*-NN, *k*-nearest neighbor; AD, Alzheimer's disease; HC, healthy controls; AD-MCI, mild cognitive impairment at time of scan with conversion to AD within 3 years; FA, fractional anisotropy; MD, mean diffusivity.
