# Supplementary material for: Prediction of Alzheimer's disease using individual structural connectivity networks
Source: Neurobiol Aging. 2012 Dec;33(12):2756–65. doi: 10.1016/j.neurobiolaging.2012.01.017 (PMC3778749; doi:10.1016/j.neurobiolaging.2012.01.017)
Supplement: Supplementary Data [file mmc1.doc]

**Supplemental Information**

**1. Anatomical atlas**

For grey matter parcellation, the whole cerebral cortex of each participant was segmented into 96 cortical regions via Harvard-Oxford human brain atlas (<http://www.fmrib.ox.ac.uk/fsl>). This anatomical atlas is a probabilistic population-based atlas; cortical regions were thresholded in a way that only voxels, which are estimated above 35% probability of being in that structure, are included in the mask. Table S1 illustrates names of used sub-regions and their corresponding consequential numbers.

*Table S1. Decoding anatomical labels.*

| ID | Cortical Regions | ID | Name |
| --- | --- | --- | --- |
| 1 | Frontal Pole | 25 | Parahippocampal Gyrus (anterior) |
| 2 | Insular Cortex | 26 | Parahippocampal Gyrus (posterior) |
| 3 | Superior Frontal Gyrus | 27 | Temporal Fusiform Cortex (anterior) |
| 4 | Middle Frontal Gyrus | 28 | Temporal Fusiform Cortex (posterior) |
| 5 | Inferior Frontal Gyrus, pars triangularis | 29 | Temporal Occipital Fusiform Cortex |
| 6 | Inferior Frontal Gyrus, pars opercularis | 30 | Planum Polare |
| 7 | Precentral Gyrus | 31 | Heschl's Gyrus (includes H1 and H2) |
| 8 | Frontal Medial Cortex | 32 | Planum Temporale |
| 9 | Juxtapositional Lobule Cortex | 33 | Postcentral Gyrus |
| 10 | Subcallosal Cortex | 34 | Superior Parietal Lobule |
| 11 | Paracingulate Gyrus | 35 | Supramarginal Gyrus (anterior) |
| 12 | Cingulate Gyrus (anterior) | 36 | Supramarginal Gyrus (posterior) |
| 13 | Frontal Orbital Cortex | 37 | Angular Gyrus |
| 14 | Frontal Operculum Cortex | 38 | Cingulate Gyrus (posterior) |
| 15 | Central Opercular Cortex | 39 | Precuneous Cortex |
| 16 | Temporal Pole | 40 | Cuneal Cortex |
| 17 | Superior Temporal Gyrus (anterior) | 41 | Parietal Operculum Cortex |
| 18 | Superior Temporal Gyrus (posterior) | 42 | Lateral Occipital Cortex (superoir) |
| 19 | Middle Temporal Gyrus (anterior) | 43 | Lateral Occipital Cortex (inferior) |
| 20 | Middle Temporal Gyrus (posterior) | 44 | Intracalcarine Cortex |
| 21 | Middle Temporal Gyrus (temporooccipital) | 45 | Lingual Gyrus |
| 22 | Inferior Temporal Gyrus (anterior) | 46 | Occipital Fusiform Gyrus |
| 23 | Inferior Temporal Gyrus (posterior) | 47 | Supracalcarine Cortex |
| 24 | Inferior Temporal Gyrus (temporooccipital) | 48 | Occipital Pole |

Here only cortical regions of left hemisphere are displayed, where IDs (1-15) indicate the Frontal Lobe (FL), IDs (16-32) the Temporal Lobe (TL), IDs (33-41) the Parietal Lobe (PL) and IDs (42-48) the Occipital Lobe (OL).

**2. Structural connectivity matrices reflect across group members averaged ISCNs**

After cortical parcellation and white matter tractography, the individual structural connection network ISCN for each individual was obtained. These ISCNs were then averaged for each group and connection attribute, and represented by corresponding structural connectivity matrices of Figures 2-4 and S1. Concerning these figures, each element of a shown matrix represents the connection between two cortical regions (Fig. S1, detailed decoding of regions in Table S1). Different groups are separately shown in the upper and lower triangular matrix. Take Fig. S1 as an example, the upper triangle matrix indicates the averaged structural connectivity for patients with mild AD and lower triangle matrix indicates the averaged structural connectivity for healthy controls. Black dots indicate that there is no connection for any subject of the group. Red to yellow dots indicate the average value of connection attribute (here, fractional anisotropy) across all subjects of the group (yellow indicates higher scores). Please note that connection attribute for FA is defined by the mean of FA-values across all voxels of fibers constituting the connection in one subject.


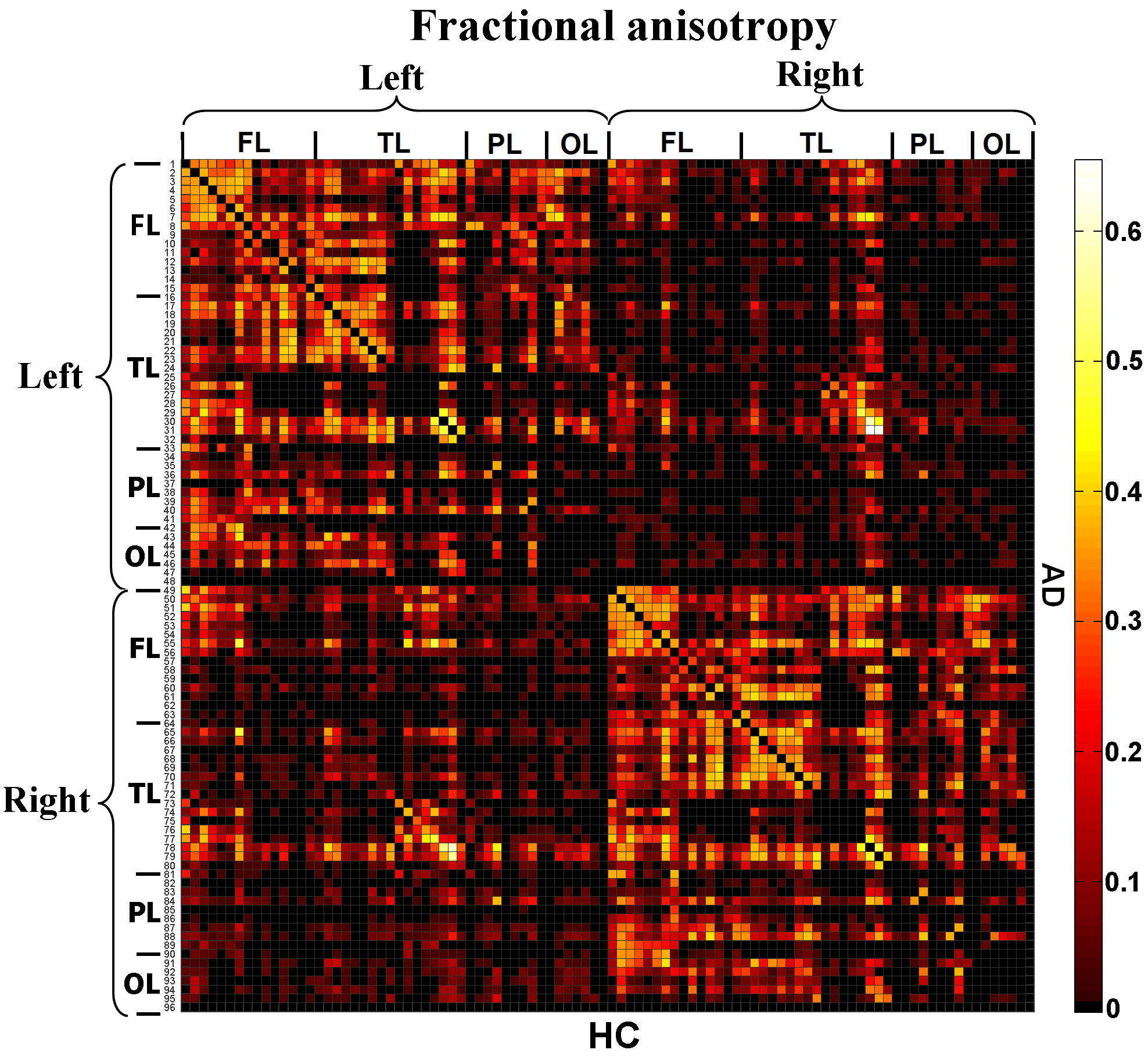


*Figure S1.* Structural brain connectivity matrix for patients with mild AD and healthy controls (HC) based on the attribute of fractional anisotropy FA. For explanation please see in the text above. FL frontal, TL temporal, PL parietal, OL occipital lobe.

**3. Feature Selection**

After construction of ISCNs, the most distinctive connections among groups were identified by feature selection. For each ISCN type, all possible group comparisons, and each round of 10-fold cross-validation and leave-one-out validation of results, respectively, Information Gain criterion was used to rate the information-based interestingness of each connection and attribute for pattern classification. For a given attribute *A* with respect to the class attribute *C*, the Information Gain is the reduction in uncertainty about the value of *C* when *A* is known. Uncertainty about class *C* is measured by its entropy *H(C)*, which is defined as:

$H(C)=\sum_{c\in C} p(c)\log_{2}p(c)$ (S1)

where *p(c)* denotes the probability of class *c*.

The entropy of the class *C* after known the attribute *A* is given as:

$H(C|A)=-\sum_{a\in A} p(a)\sum_{c\in C} p(c|a)\log_{2}p(c|a)$ (S2)

where *p*(*c|a*) is the conditional probability of *c* for a given *a.*

Since the reduction amount of the entropy of the class after knowing the attribute *A* reflects the additional information about the class provided by the attribute, Information Gain captures the importance of attribute for the class concept. Formally, the Information Gain *IG(A)* is defined as:

*IG*(*A*) *= H*(*C*) – *H*(*C|A*) (S3)

In order to find most distinguishing connections among different groups, therefore, the discriminative power of each connection for group comparison was measured by the information gain. Since three attributes (fiber density, FA and MD) were used to characterize each connection, we considered the distinguishing connections for each attribute and each group comparison separately, resulting in 9 different patterns of selected connections (indicated by green dots of Fig 2-4; Table S2, S3). These selected connections with different attributes will separately enter into three typical classifiers for prediction.

*Table S2. The absolute number and percentage of selected connections among group comparison via Information Gain-based feature selection corresponding to Figures 2-4.*

|  | Fiber density | FA | MD |
| --- | --- | --- | --- |
| Mild AD vs HC | 53 (1.2%) | 56(1.2%) | 119(2.6%) |
| Mild AD vs. AD-MCI | 88(1.9%) | 56(1.2%) | 81(1.8%) |
| AD-MCI vs. HC | 34(0.7%) | 26(0.6%) | 33(0.7%) |

AD Alzheimer’s disease; HC healthy controls; MCI mild cognitive impairment; AD-MCI MCI at baseline with conversion to AD within 3 years; FA fractional anisotropy; MD mean diffusivity

**4. ISCN: Modulation of validation, classification, and connection definition**

*Table S3. Classification accuracy for individual structural connectivity networks using one-leave-out cross validation.*

SVM support vector Machine; *k*-NN *k*-nearest neighbor; AD Alzheimer’s disease; HC healthy controls; MCI mild cognitive impairment; AD-MCI MCI at baseline with conversion to AD within 3 years; FA fractional anisotropy; MD mean diffusivity

|  | SVM | *k*-NN | Naive Bayes |
| --- | --- | --- | --- |
| Mild AD vs HC |  |  |  |
| Fiber density | 100.0% | 86.84% | 97.37% |
| FA | 89.47% | 92.11% | 100.0% |
| MD | 97.37% | 92.11% | 92.11% |
| Mild AD vs. AD-MCI |  |  |  |
| Fiber density | 87.50% | 75.00% | 92.50% |
| FA | 80.00% | 80.00% | 85.00% |
| MD | 85.00% | 82.50% | 87.50% |
| AD-MCI vs. HC |  |  |  |
| Fiber density | 97.73% | 65.91% | 95.45% |
| FA | 88.64% | 88.64% | 97.73% |
| MD | 93.18% | 84.09% | 100.0% |

*Table S4. Classification accuracies with the threshold of 3 fibers using 10-fold cross-validation and leave-one-out cross-validation (LOOCV) respectively.*

|  | SVM | *k*-NN | Naive Bayes |
| --- | --- | --- | --- |
| Mild AD vs HC |  |  |  |
| Fiber density | 97.37% (100.0%) | 89.47% (86.84%) | 97.37% (97.37%) |
| FA | 86.84% (89.47%) | 84.21% (86.84%) | 89.47% (92.11%) |
| MD | 92.11% (86.84%) | 81.58% (76.32%) | 89.47% (89.47%) |
| Mild AD vs. AD-MCI |  |  |  |
| Fiber density | 82.50% (80.00%) | 72.50% (77.50%) | 90.00% (92.50%) |
| FA | 70.00% (75.00%) | 67.50% (62.50%) | 75.00% (82.50%) |
| MD | 77.50% (82.50%) | 80.00% (72.50%) | 82.50% (87.50%) |
| AD-MCI vs. HC |  |  |  |
| Fiber density | 100.0% (97.73%) | 75.00% (70.45%) | 97.73% (97.73%) |
| FA | 84.09% (81.82%) | 79.55% (77.27%) | 86.36% (88.64%) |
| MD | 86.36% (90.91 %) | 77.27% (72.73%) | 90.91% (86.36%) |

SVM support vector machine; *k*-NN *k*-nearest neighbor; AD Alzheimer’s disease; HC healthy controls; AD-MCI mild cognitive impairment at time of scan with conversion to AD within 3 years; FA fractional anisotropy; MD mean diffusivity.

*Table S5. Classification accuracies with the threshold of 5 fibers using 10-fold cross-validation and leave-one-out cross-validation (LOOCV) respectively.*

|  | SVM | *k*-NN | Naive Bayes |
| --- | --- | --- | --- |
| Mild AD vs HC |  |  |  |
| Fiber density | 100.0% (97.37%) | 84.21% (81.58%) | 97.37% (97.37%) |
| FA | 92.11% (89.47%) | 92.11% (94.74%) | 92.11% (92.11%) |
| MD | 89.47% (92.11%) | 81.58% (81.58%) | 94.74% (97.37%) |
| Mild AD vs. AD-MCI |  |  |  |
| Fiber density | 82.50% (80.00%) | 75.00% (77.50%) | 90.00% (92.50%) |
| FA | 90.00% (92.50%) | 75.00% (77.50%) | 80.00% (90.00%) |
| MD | 67.50% (65.00%) | 67.50% (65.00%) | 80.00% (80.00%) |
| AD-MCI vs. HC |  |  |  |
| Fiber density | 95.45% (97.73%) | 75.00% (63.64%) | 93.18% (100.0%) |
| FA | 93.18% (97.73%) | 86.36% (93.18%) | 93.18% (95.45%) |
| MD | 86.36% (88.64%) | 86.36% (88.64%) | 93.18% (93.18%) |

SVM support vector machine; *k*-NN *k*-nearest neighbor; AD Alzheimer’s disease; HC healthy controls; AD-MCI mild cognitive impairment at time of scan with conversion to AD within 3 years; FA fractional anisotropy; MD mean diffusivity.

**5. Most informative connections underlying group differences**


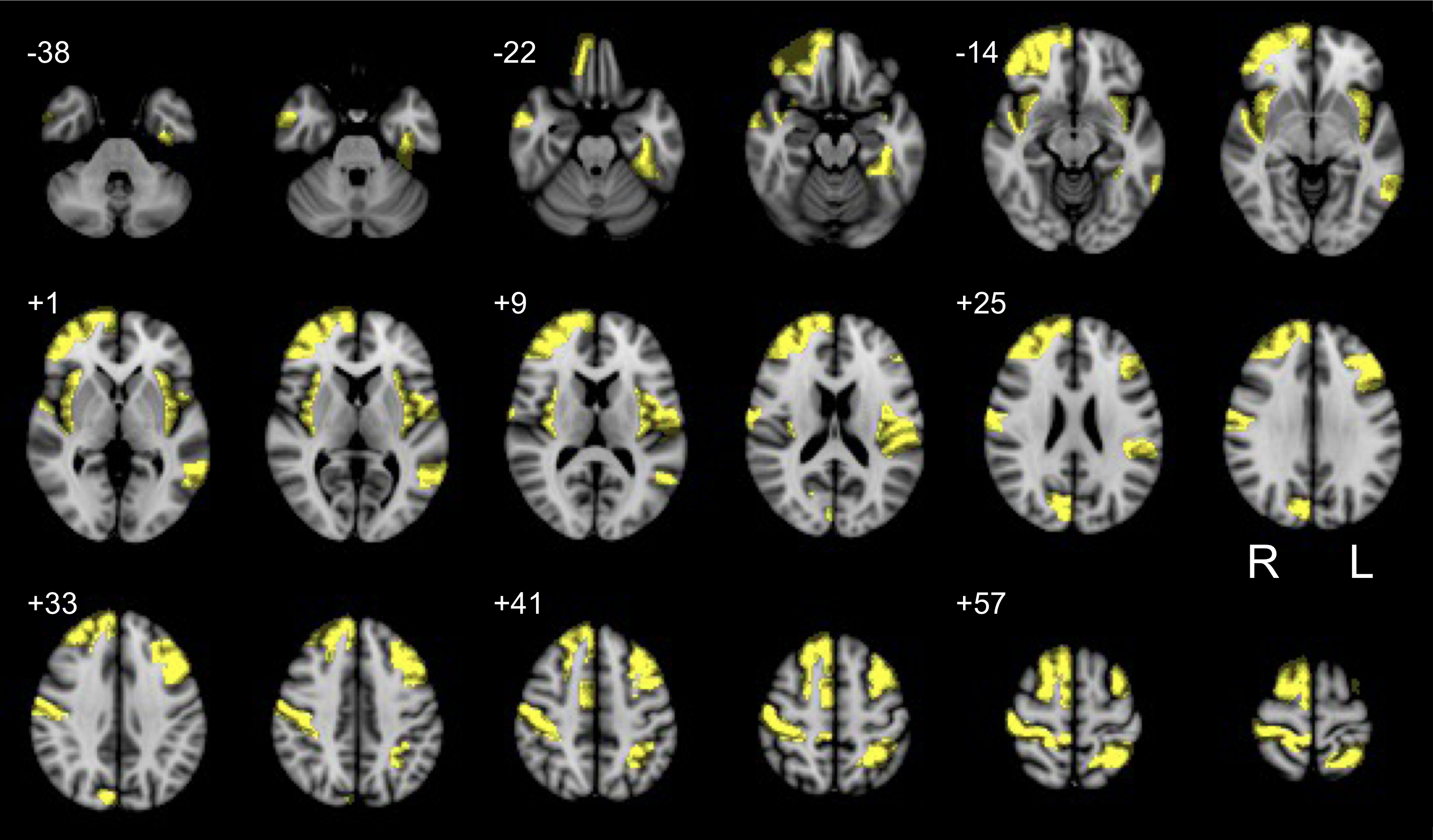


*Figure S5. Most informative features in distinguishing both AD and AD-MCI from HC.* Colored areas are the endpoints of connections with positive information gain for both patient groups (AD and AD-MCI), compared to healthy controls (HC). To arrive at this set of areas, we first sought out connections with positive information gain for all three ISCN types (fiber density, FA, and MD), separately for the comparisons AD vs. HC and AD-MCI vs. HC. Endpoints of connections showing up in both group comparisons are represented here. Regions (yellow, taken from the Harvard-Oxford cortical atlas) are rendered on axial slices of a standard brain. Numbers indicate Z coordinates in MNI space. Note that the right hemisphere is displayed on the left side of each slice.

*Table S6.* Endpoints of connections with consistent positive information gain across ISCN types and patient-control group comparisons.

| Hemisphere | Region | Hemisphere | Region |
| --- | --- | --- | --- |
| L | Frontal Pole | R | Insula |
| L | Insula | R | Middle Frontal Gyrus |
| L | Superior Frontal Gyrus | R | Posterior Middle Temporal Gyrus |
| L | Anterior Middle Temporal Gyrus | R | Superior Parietal Lobule |
| L | Postcentral Gyrus | R | Posterior Temporal Fusiform Gyrus |
| L | Juxtapositional Lobule / Supplementary Motor Cortex | R | Central Operculum |
| L | Cuneus | R | Parietal Operculum |
| L | Planum Polare |  |  |
